# Rivaroxaban versus warfarin for the management of left ventricle thrombus

**DOI:** 10.1186/s43044-021-00164-7

**Published:** 2021-05-01

**Authors:** Monirah A. Albabtain, Yahya Alhebaishi, Ola Al-Yafi, Hatim Kheirallah, Adel Othman, Haneen Alghosoon, Amr A. Arafat, Ahmed Alfagih

**Affiliations:** 1 Pharmacy Department, Prince Sultan Cardiac Center, Riyadh, Saudi Arabia; 2 Adult Cardiology Department, Prince Sultan Cardiac Center, Riyadh, Saudi Arabia; 3 Clinical Pharmacy Department, Al Maarefa College, Riyadh, Saudi Arabia; 4 Cardiac Research Department, Prince Sultan Cardiac Center, Riyadh, Saudi Arabia; 5 Adult Cardiac Surgery Department, Prince Sultan Cardiac Center, Riyadh, Saudi Arabia; 6 Cardiothoracic Surgery Department, Tanta University, Tanta, Egypt

**Keywords:** Left ventricle thrombus, Non-vitamin K dependent oral anticoagulant rivaroxaban, Warfarin

## Abstract

**Background:**

Rivaroxaban has been recently introduced for the management of non-valvular intra-cardiac thrombosis with variable results. We aimed to compare the results of the off-label use of rivaroxaban versus warfarin in the management of patients with left ventricle (LV) thrombus. This research is a retrospective study conducted on 63 patients who had LV thrombus from January to December 2016. We compared patients treated with warfarin (*n*=35) to patients who had rivaroxaban (*n*=28), and study outcomes were time to thrombus resolution, bleeding, stroke, and mortality.

**Results:**

The median duration of treatment was 9.5 (25th-75th percentiles: 6-32.5) months for rivaroxaban and 14 (3-41) months for warfarin. Thrombus resolution occurred in 24 patients in the warfarin group (68.6%) and 20 patients in the rivaroxaban group (71.4%). The median time to resolution in the warfarin group was 9 (4-20) months and 3 (2-11.5) months in the rivaroxaban group. Thrombus resolution was significantly faster in patients on rivaroxaban (*p*= 0.019). Predictors of thrombus resolution were thrombus surface area (HR: 1.21; CI 95% (1.0-1.46); *p*= .048) and the use of rivaroxaban (HR: 1.92; CI 95% (1.01-3.65); *p*= 0.048). There was no difference in stroke, bleeding, and mortality between both groups.

**Conclusion:**

Rivaroxaban was as effective and safe as warfarin in managing patients with left ventricle thrombus. Larger randomized clinical trials are recommended to confirm our findings.

## Background

Intracardiac thrombus is a potentially life-threatening condition with a high risk of embolic complications [1]. The traditional anticoagulant for intracardiac thrombus is vitamin K antagonists. However, they were replaced recently in specific conditions with direct oral anticoagulants (DOAC) [2]. DOACs have several advantages over warfarin, including predictable kinetics and no need for continuous monitoring [3].

The AHA/ACC guidelines [4] recommended the use of rivaroxaban as an alternative to warfarin in patients with left ventricle (LV) thrombus who are intolerant to warfarin therapy. However, the use of rivaroxaban for the primary management of LV thrombus was not thoroughly investigated [5, 6]. This study’s objective was to compare the results of the off-label use of rivaroxaban versus warfarin in managing patients with LV thrombus.

## Methods

### Study design and patients

This retrospective cohort study included patients with LV thrombus in association with myocardial infarction (MI) or heart failure/cardiomyopathy admitted between January and December 2016. We included patients with LV thrombus documented by echocardiography within 1 week before starting treatment and the absence of contraindication to anticoagulation. Patients with active bleeding and severe liver or renal impairment were excluded from the study. Patients on a non-steroidal anti-inflammatory drug or a potent inhibitor or inducer of cytochrome P450 and pregnant ladies were not included.

**Fig. 1:**
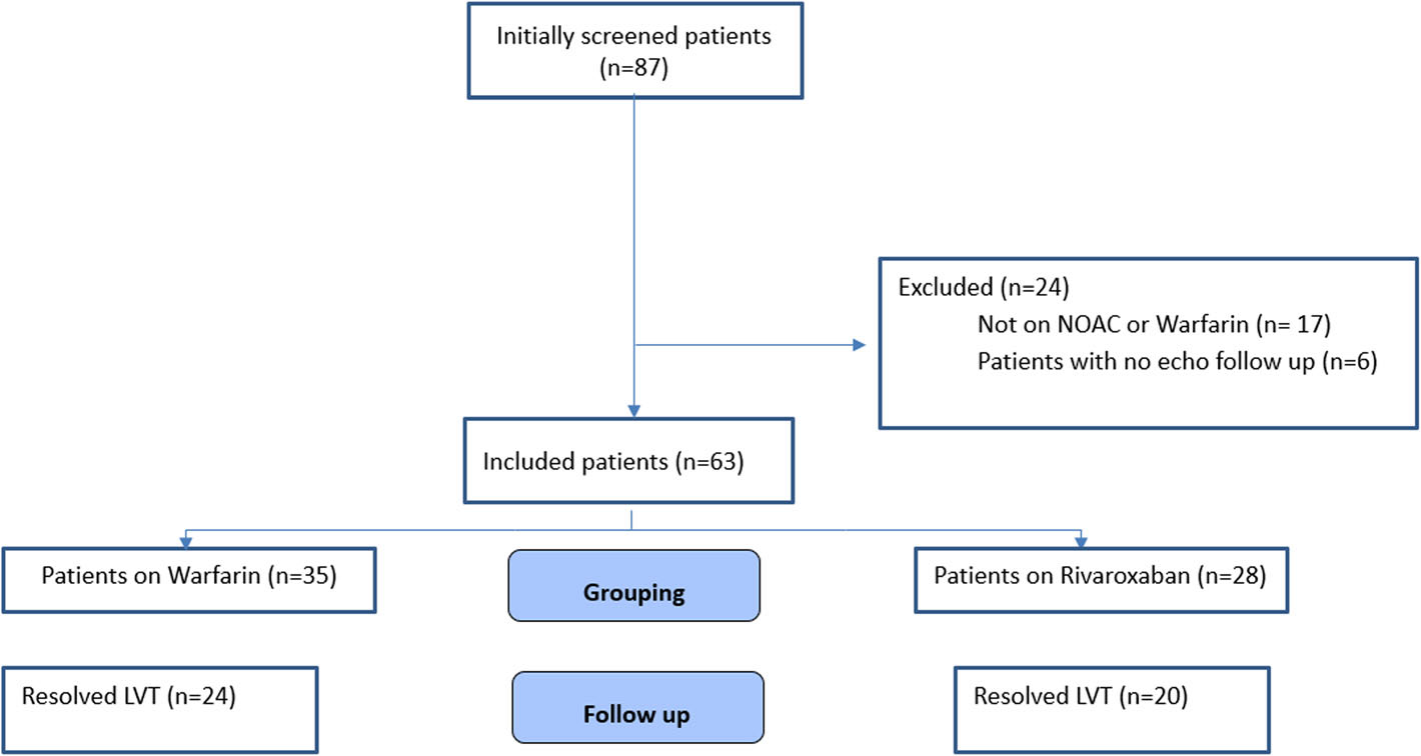
Study flowchart (LVT, left ventricular thrombus)

During the study period, 87 patients had LV thrombus; 63 of them were included in our study. We grouped the patients into two groups: the warfarin group (*n*= 35) and the rivaroxaban group (*n*= 28). The study flowchart was presented in Fig. 1.

The study was approved by the Institutional Review Board (Reference number E16012), and the need for patients’ consent was waived.

### Treatment and follow-up protocol

Rivaroxaban was administrated in a dose of 20 mg per os (PO) daily based on creatinine clearance upon diagnosis of LV thrombus. Five patients received an adjusted rivaroxaban dose of 15 mg due to renal insufficiency. Patients on warfarin were followed in the anticoagulation clinic with a target international normalization ratio (INR) of 2-3. All patients on warfarin were closely monitored and achieved therapeutic INR levels during the study period.

We used Phillips iE33 echocardiography machines for the diagnosis and follow-up of the LV thrombus. Standard images were obtained as per the American Society of Echocardiography protocol, in addition to zoom-in images to enhance the thrombus visibility. We did not use LV contrast in any patient. Thrombus surface area was measured and reported. As the thrombus is a 3D structure, a one-dimensional measurement may not reflect the size, especially for thrombi, which had irregular contour. Therefore, we opted to measure the thrombus in 2 dimensions, and the multiplication of the two figures was approximate of the surface area. Patients had follow-up echocardiography every 2 months or at the discretion of the treating physician. There were 279 echocardiographic studies available for all patients. Patients were followed for bleeding events using the Bleeding Academic Research Consortium (BARC) definition [7].

### Study endpoints

We compared patients who had warfarin (*n*=35) to patients who had rivaroxaban (*n*=28), and study outcomes were time to thrombus resolution, bleeding, stroke, and mortality.

### Statistical analysis

Continuous variables were presented as mean and standard deviation (SD) if normally distributed and compared with the *t* test or median (25th-75th percentiles) if non-normally distributed and compared using the Mann-Whitney test. Categorical variables were presented as numbers and percentages and compared using the chi-square test or Fisher exact test if the expected frequency was less than 5. The time to events variables were compared using the Log-rank test. Multivariable Cox regression was used to identify factors affecting time to thrombus resolution. We performed the intention to treat analysis to simulate clinical trials. All statistical analysis was performed using Stata IC 16 (Stata Corp, College Station, Texas, USA).

## Results

### Baseline data

Patients’ characteristics were presented in Table 1. Males presented 97% of patients in the warfarin group and 85.7% in the rivaroxaban group. All patients were on beta-blockers, spironolactone, and angiotensin-converting enzyme inhibitors (ACEI) or angiotensin receptor blockers (ARBs). There was no difference in the distribution of aspirin and p2Y12 inhibitors between the groups (Table 1).

**Table 1:** Comparison of baseline data between warfarin and rivaroxaban groups

	Warfarin (*n*= 35)	Rivaroxaban (*n*= 28)	*p*
Age (years)	59 (15.62)	58.25 (17.73)	0.86
Male	34 (97.14%)	24 (85.71%)	0.16
BMI (kg/m^2^)	28.1 (4.04)	27.59 (5.88)	0.69
BSA (m^2^)	1.91 (0.16)	1.87 (0.25)	0.51
Diabetes mellitus	16 (45.71%)	12 (42.86%)	0.82
Hypertension	19 (54.29%)	13 (46.43%)	0.54
Atrial fibrillation	2 (5.71%)	1 (3.57%)	˃ 0.99
Myocardial infarction	25 (71.43%)	16 (57.14%)	0.24
Hemoglobin (g/dl)	14.1 (1.91)	13.4 (2.25)	0.19
Creatinine clearance (ml/min)	80.6 (38.66)	92.28 (32.37)	0.21
ALT (U/L)	21 (17-34)	31 (18-43)	0.10
ALP (U/L)	90 (70-108)	86 (72-115)	0.97
Aspirin	20 (57.14%)	19 (67.86%)	0.38
P2Y12 inhibitors (clopidogrel)	21 (60%)	13 (46.43%)	0.24
Ejection fraction (%)	27.29 (7.8)	26.43 (8.15)	0.67
LVT surface area (cm^2^)	1.77 (0.66-2.21)	1.75 (0.7-3.49)	0.3
Moderate MR	5 (14.3%)	7 (25%)	0.28
Moderate TR	3 (8.6%)	5 (17.9%)	0.45
Dilated LV	9 (25.71%)	14 (50%)	0.047
INR base	1.2 (1.1-2.2)	1.2 (1.1-1.6)	0.92

*ALP* alkaline phosphatase, *ALT* alanine transaminase, *BMI* body mass index, *BSA* body surface area, *INR* international normalization ratio, *LVT* left ventricular thrombus, *MR* mitral regurgitation, *TR* tricuspid regurgitation

Continuous data are presented as mean (standard deviation) if normally distributed and median (Q1-Q3) if non-normally distributed and categorical data as number (%)

Patients on aspirin or P2Y12 inhibitors had these medications on admission and continued during warfarin or rivaroxaban therapy

Aspirin dose was 81 mg/day; clopidogrel dose was 75 mg/day

There was no difference in the LV thrombus’ surface area before starting treatment between the two groups; however, patients on rivaroxaban had significantly dilated LV.

### Study outcomes

**Fig. 2:**
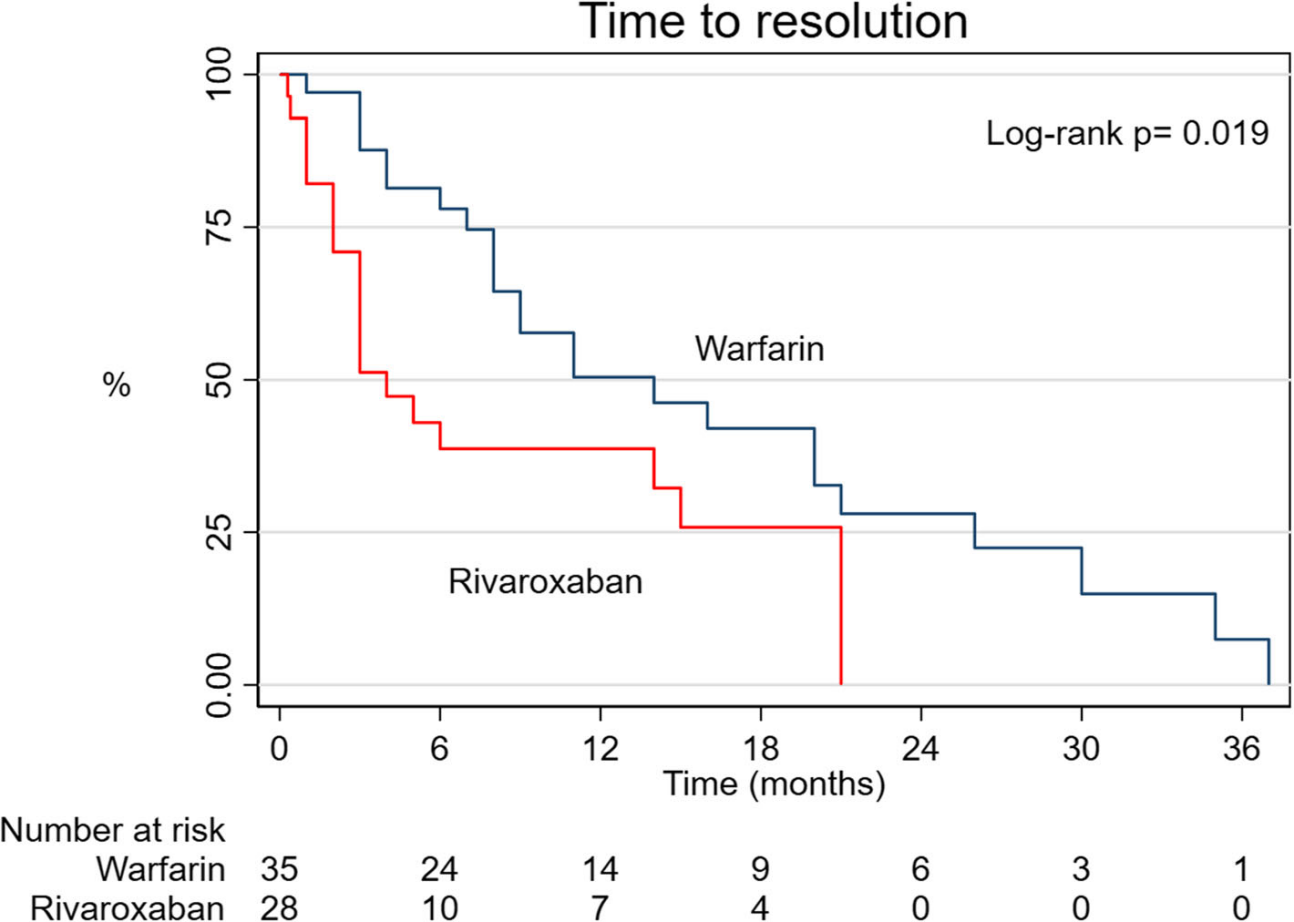
Kaplan-Meier curve to time to resolution of the left ventricular thrombus in warfarin and rivaroxaban groups

The median duration of treatment was 9.5 (6-32.5) months for rivaroxaban and 14 (3-41) months for warfarin. One patient was shifted to warfarin because of recurrent transient ischemic attacks (TIA). Three patients on warfarin were turned to rivaroxaban (8.57%); 2 had AF, and one patient had persistent LV thrombus. Thrombus resolution occurred in 24 patients in the warfarin group (68.57%) and 20 patients in the rivaroxaban group (71.43%). The median time to resolution in the warfarin group was 9 (4-20) months and 3 (2-11.5) months in the rivaroxaban group. Thrombus resolution was significantly shorter in patients on rivaroxaban *p*= 0.019 (Fig. 2).

Predictors of thrombus resolution were thrombus surface area (HR: 1.21; CI 95% (1.0-1.46); *p*= .048) and the use of rivaroxaban (HR: 1.92; CI 95% (1.01-3.65); *p*= 0.048) (Table 2).

**Table 2:** Multivariable analysis of predictors of left ventricular thrombus resolution

	HR (95% CI)	*p* value
Rivaroxaban	1.92 (1.01-3.65)	0.048
Baseline thrombus surface area	1.21 (1.0-1.46)	0.048
Body surface area	0.36 (0.06-2.27)	0.275
History of myocardial infarction	0.75 (0.39-1.42)	0.372

Stroke occurred in one patient in the warfarin group (2.86%) before thrombus resolution and one patient in the rivaroxaban group (3.75%) after thrombus resolution (Log-rank *p* ˃0.99). Bleeding occurred in one patient in the warfarin group (2.86%) and two patients in the rivaroxaban group (7.14%) (Log-rank *p*= 0.23). We did not report recurrence during the follow-up period.

One patient had peripheral emboli in the rivaroxaban group (3.75%) versus no patient in the warfarin group. No difference in mortality was found between the two groups (warfarin *n*=3; 8.57%, rivaroxaban *n*= 2; 7.14%) (Log-rank *p*= 0.64).

## Discussion

This retrospective study was conducted on 63 patients with left ventricle thrombus treated with rivaroxaban or warfarin. Time to resolution was shorter with rivaroxaban with no difference in the resolution rate and complications between groups.

The use of rivaroxaban in the management of left ventricle thrombus was reported in several conditions. Rivaroxaban was used to manage a patient with tachycardia-induced heart failure and failed warfarin therapy [8], and patients with poor anticoagulation quality, and the reported outcomes were good [9]. Left ventricle thrombus complicating myocardial infarction was treated with rivaroxaban combined with dual antiplatelet therapy, and thrombus resolution was reported in a variable time from 1 to 3 months [10]. Complete dissolution of thrombus within 2 to 4 weeks in patients with acute coronary syndrome after the percutaneous coronary intervention was reported [1]. The median duration of rivaroxaban treatment for the resolution of the left ventricle thrombus in a meta-analysis of 29 patients was 30 days [10]. We did not find a difference in the number of patients who had thrombus resolution in the rivaroxaban vs. warfarin group. However, the time to resolution was shorter in patients who had rivaroxaban.

Data on direct oral anticoagulants, specifically in patients with intra-cardiac thrombus, are limited [11]. In a randomized trial, rivaroxaban was more effective than warfarin in the resolution of left atrial thrombus in patients with non-valvular atrial fibrillation [12].

In a recent cohort study, warfarin (*n*= 300) was compared with DOAC (*n*= 185) to manage LV thrombus. The risk of ischemic stroke and systemic emboli was higher with DOAC [13]. The difference in the outcomes between this study and our research could be attributed to the different sample sizes that could not detect a difference in complication rate. Additionally, we included patients who had rivaroxaban only, and the response to various DOACs could differ.

The rate of thrombus resolution was evaluated in scarce studies. Jones and associates reported a higher LV thrombus resolution rate in patients who received DOAC than warfarin in a study of 101 patients who had LV thrombus after acute myocardial infarction in 3 years [14]. Moreover, they reported a higher rate of bleed with DOAC and no difference in thromboembolic complications [14]. On the other hand, Bass and colleagues reported higher blood transfusion requirements in patients who received warfarin than DOAC and no difference in other outcomes [15].

In a study on DOAC therapy in patients with left ventricle thrombus, gastrointestinal bleeding requiring transfusion with reported in 2/17 patients [16]. Our patients were screened for bleeding according to the BARC definition, and two patients had bleeding in the rivaroxaban group compared to one patient in the warfarin group. The initial reports on the use of rivaroxaban for the management of left ventricle thrombus are encouraging. It showed a high efficacy with few side effects. One of the potential advantages of rivaroxaban is that it does not need monitoring. Three patients were shifted to rivaroxaban, which was indicated due to poor INR control in those patients.

The efficacy of warfarin was assessed with time in the therapeutic range (TTR) in several studies [17]. Maniwa and colleagues found that appropriate anticoagulation treatment confirmed with TTR could reduce embolism risk without increasing bleeding risk [18]. We did not study this relation in our study as the sample size is too small to generate sufficient TTR to compare. In a study by Sumaya and associates, delayed thrombus lysis was associated with worse outcomes [19]. We did not find this relation in our study, which could be attributed to the small sample size. However, there is a potential advantage of rivaroxaban in those patients since it is associated with the faster resolution, which could decrease the adverse events if used on a wide scale.

Our study had several limitations, including the small patients’ number, which is accepted for this new off-label indication of rivaroxaban. The sample size was sufficient to detect a difference in time to thrombus resolution; however, the lack of significant difference in complication rates could be an issue. The study is a retrospective research with its inherent selection and referral biases. However, we reported faster resolution of LV thrombus with DOAC, which is under-reported in the literature. The study highlighted rivaroxaban’s safety in patients with LV thrombus, and further randomized trials are warranted.

## Conclusion

Rivaroxaban might be as effective and safe as warfarin in managing patients with left ventricle thrombus with shorter LV thrombus resolution time. However, the study is limited by the sample size, and larger randomized clinical trials are recommended to confirm our findings.

## Data Availability

Upon reasonable requests

## Abbreviations

ACEi: Angiotensin-converting enzyme inhibitors
ARBs: Angiotensin receptor blockers
BARC: Bleeding Academic Research Consortium
DOAC: Direct oral anticoagulants
LV: Left ventricle
